# Diagnostic accuracy study of the multiplex Truenat MTB Ultima/COVID-19 assay for simultaneous detection of Tuberculosis and SARS-CoV2 (COVID-19)

**DOI:** 10.1371/journal.pgph.0005859

**Published:** 2026-06-05

**Authors:** Hafsah Tootla, Rita Székely, James Sserubiri, Widaad Zemanay, Mala Patidar, Moses Joloba, Cesar Ugarte-Gil, Morten Ruhwald, Manju Purohit, Helen Cox, Carlos Zamudio, Willy Ssengooba, Adam Penn-Nicholson, on behalf of the COMBO study team

**Affiliations:** 1 Division of Medical Microbiology, Faculty of Health Sciences, University of Cape Town, Cape Town, South Africa,‌‌; 2 Department of Medical Microbiology, National Health Laboratory Service, Groote Schuur Hospital, Cape Town, South Africa; 3 FIND, Geneva, Switzerland; 4 Department of Medical Microbiology Makerere University, Kampala, Uganda; 5 Central Research Laboratory, RuxmaniBen Deepchand Gardi Medical College, Ujjain, India; 6 Instituto de Medicina Tropical Alexander von Humboldt, Universidad Peruana Cayetano Heredia, Lima, Peru; 7 Department of Epidemiology, The University of Texas Medical Branch, Galveston, Texas, United States of America; 8 Department of Public Health Sciences, Karolinska Institutet, Stockholm, Sweden; 9 Burnet Institute, Melbourne, Australia; Imperial College London / North Bristol NHS Trust / University of Heidelberg, UNITED KINGDOM OF GREAT BRITAIN AND NORTHERN IRELAND

## Abstract

The COVID-19 pandemic led to significantly disrupted tuberculosis case detection and management. Additionally, overlapping symptoms, radiological findings and risk factors make differentiating tuberculosis and COVID-19 disease difficult. We conducted a prospective multicentre diagnostic accuracy study to determine sensitivity, specificity and operational characteristics of the Truenat MTB Ultima/COVID-19 assay, using a combined sputum plus nasopharyngeal swab specimen, in a single multiplex molecular assay. Participants were consenting adults with presumptive tuberculosis enrolled via convenience sampling from Uganda, Peru, South Africa, and India between August 2022 and December 2023. A microbiological reference standard of sputum GeneXpert MTB/RIF Ultra and culture was used for tuberculosis, whereas national RT-PCR was used for COVID-19. Wilson’s score method was used to determine the sensitivity and specificity. Of 1,928 participants enrolled, median age was 38 years (IQR 28–50), 359/1928 (18.6%) previously had TB, and 287/1928 (14.9%) were HIV-positive. Overall prevalence of tuberculosis was 24.8% [95% CI 22.9-26.8%]. Prevalence of COVID-19 was 3.8% [3.1-4.8%] overall, and 4.7% [3.1-7.0%] in those with confirmed tuberculosis. Overall sensitivity of Truenat MTB Ultima/COVID-19 for tuberculosis was 79.8% [95% CI 76.0-83.2%]. When comparing paired samples, Truenat MTB Ultima/COVID-19 had a 9.5% [6.5-13.1%] decreased sensitivity against sputum TB culture, compared to GeneXpert Ultra; (82.8% [78.9-86.1%] vs 92.4% [89.4-94.5%]). Overall specificity of Truenat MTB Ultima/COVID-19 for tuberculosis was 98.9% [98.2-99.3%]. For COVID-19 detection, sensitivity of Truenat MTB Ultima/COVID-19 was 64.4% [52.9-74.4%], with specificity of 99.2% [98.7-99.5%]. Although optimal diagnostic performance was not demonstrated, the potential and need for rapid development of tests that integrate tuberculosis diagnosis with detection of other relevant respiratory infections is highlighted. The study was registered on ClinicalTrials.gov (NCT05405296).

## Introduction

For many decades, tuberculosis (TB) has been the leading cause of infectious death, temporarily surpassed by the SARS-CoV2 (COVID-19) virus during the COVID-19 pandemic, and now most recently re-emerging once again as the number one cause of death by infectious disease [1,2].

Intensified research and innovation in TB control, with a focus to meet the WHO End TB strategy targets (by the year 2030) of 90% reduction in TB deaths, 80% reduction in TB incidence rate, and no TB-affected families facing catastrophic costs due to TB, have called for integrated person-centred TB care and have resulted in the development of revolutionary rapid molecular TB diagnostic tests, and shorter effective oral treatment and preventative strategies [3]. Despite these powerful advancements, major gaps, particularly in TB case detection (including affordability and accessibility) and treatment coverage, still exist [1,3]. Over 10 million people per year still develop TB disease and ending the TB epidemic remains an urgent priority [1,3,4]. Although substantial progress had been made with TB control in the last decade, the COVID-19 pandemic caused significant setbacks [1,4–6]. TB case detection dropped by ~20%, access to TB services during the pandemic was limited and there was an overall ~20% reduction in the number of people receiving TB preventative therapy [5–7]. Tuberculosis prevalence amongst hospitalised COVID-19 cohorts was estimated at between 2–8% in high TB-burden settings and overlapping clinical symptoms, radiological features and onset of symptoms, particularly in TB-endemic countries, made differentiating TB and COVID-19 disease difficult [7–10]. Furthermore, shared risk factors for TB and COVID-19 infection and disease, such as overcrowded living spaces and comorbidities like HIV and diabetes, as well as co-infection with both TB and COVID-19, added complexity in making an accurate diagnosis of TB when resources were being redirected towards diagnosis and containment of COVID-19 [5].

The COVID-19 pandemic brutally demonstrated the consequences of disrupted TB diagnosis and care, and highlighted the need for rapid integrated testing strategies to diagnose and limit TB transmission, even when new threats like the COVID-19 pandemic occur.

The development of the Truenat MTB Ultima/COVID-19 multiplex PCR was in response to the unmet need for a single test capable of diagnosing both important high morbidity and high mortality infectious diseases. We conducted a multi-country study to determine the diagnostic accuracy of the Truenat MTB Ultima/COVID-19 assay for the detection of both TB and COVID-19 against TB and COVID-19 microbiological reference standards (MRS) respectively, and against a TB comparator assay currently in use in each country.

## Materials and methods

### Study design and population

This prospective multinational diagnostic accuracy study enrolled outpatient adults (age  ≥18 years) by convenience sampling of consecutively enrolled participants presenting at participating healthcare facilities in Uganda (Makerere University, Kampala) between 31 August 2022 and 05 December 2023; in Peru (Instituto de Medicina Tropical Alexander von Humboldt, Universidad Peruana Cayetano Heredia, Lima) between 26 September 2022 and 05 December 2023; in South Africa (Site B Clinic, Khayelitsha, Cape Town) between 19 October 2022 and 05 December 2023; and in India (RD Gardi Medical College, Ujjain, Madhya Pradesh) between 20 December 2022 and 05 December 2023. The sites in Peru, India and Uganda are tertiary hospitals, whereas the site in South Africa was a primary health centre facility. Once all sites had achieved their sample size target, participants were enrolled using competitive enrolment.

Participants were approached for enrolment if they reported ≥ 1 of the following symptoms suggestive of TB: cough ≥ 2 weeks, fever, night sweats or unintended weight-loss. Participants were excluded from the study if they received any prior TB treatment within 60 days of enrolment or any prior TB preventative therapy within 6 months of enrolment, were unable to provide the required first-day samples, or if all the required samples had not been collected before a 3rd dose of TB treatment was taken. Samples with indeterminate or missing test results (either index or reference) were excluded from the primary analysis.

Participants provided written informed consent, and the study received ethical approval from all participating sites (Uganda: Makerere University School of Biomedical Sciences Research Ethics Committee (MAKSBS-REC: SBS-2022–122) and the Uganda National Council for Science and Technology (UNCST#HS2346ES); Peru: Human Research Ethics Committee of the Universidad Peruana Cayetano Heredia (SIDISI 207221); India: RD Gardi Medical College IEC (IEC 05–06/2022); South Africa: Human Research Ethics Committee of the University of Cape Town (HREC 070/ 2022). The study was registered with ClinicalTrials.gov (NCT05405296), and in Peru the study was registered with the Peruvian National Institute of Health Repository PRISA (number 2573). The study protocol has been previously published [11].

Additional information regarding the ethical, cultural, and scientific considerations specific to inclusivity in global research is included in the supporting material (S1 Checklist).

### Primary and secondary outcomes

The primary outcome for this study was to determine the diagnostic accuracy of Truenat MTB Ultima/COVID-19 for TB detection compared to a defined TB microbiological reference standard (MRS).

Secondary outcomes were 1) to determine the diagnostic accuracy of Truenat MTB Ultima/COVID-19 for COVID-19 detection compared to a COVID-19 MRS, 2) to determine the diagnostic accuracy of Truenat MTB Ultima/COVID-19 for TB detection compared to GeneXpert Ultra using TB culture as the reference standard, 3) to determine the prevalence of COVID-19 amongst all study participants, 4) to determine the prevalence of COVID-19 amongst participants with confirmed TB based on a positive TB MRS, 5) to determine the diagnostic accuracy of Truenat MTB Ultima/COVID-19 for TB detection and COVID-19 detection using an alternative sample of tongue and mid-turbinate nasal swab compared to the TB and COVID-19 MRS respectively. A post-hoc objective was included to determine the proportion of non-determinate results (assay errors, invalids and indeterminate results).

### Sample collection and testing

On Day 1 of enrolment, a healthcare worker collected two nasopharyngeal swabs for COVID-19 testing with the COVID-19 MRS and Truenat MTB Ultima/COVID-19 multiplex PCR respectively. For TB testing, a minimum of 3 ml of homogenised sputum, split for testing with the Truenat MTB Ultima/COVID-19 PCR (1 ml) and TB MRS (2 ml), respectively, was collected. One healthcare-worker-collected mid-turbinate nasal swab and one healthcare-worker-collected tongue swab (the alternative and easier-to-collect sample types compared to nasopharyngeal swab and sputum) was collected for both COVID-19 and TB testing. Three additional healthcare-worker-collected tongue swabs were collected for biobanking. Baseline demographic and clinical data were captured, and diabetes and hypertension screening performed. Participants were sent home with a labelled empty sputum sample container for next day sample collection.

On Day 2 (or within 7 days of enrolment provided that all the required samples were collected before a 3^rd^ dose of TB treatment was taken), participants collected 2 ml of early morning sputum at home, and brought this to the facility for TB testing. At the facility, participants self-swabbed two tongue swabs while being observed by a healthcare worker. Thereafter two additional healthcare-worker-collected tongue swabs were also collected. All tongue swabs from Day 2 were stored for biobanking.

Biobanked samples, and where available, digital chest X-rays that were anonymised after clinician review, were collected for storage in a databank for future research and analysis.

Clinical information and reference test results were not available to laboratory staff performing the index test. Clinical information and index test results were not available to laboratory staff performing the reference tests. Index test results from this research study were not available to clinicians or used for clinical care.

### Truenat MTB Ultima/COVID-19 multiplex PCR assay description

The assay was performed according to manufacturer instructions. Briefly, 500 µl of liquified expectorated sputum was added directly to a vial containing a nasopharyngeal swab in 500 µl Molbio Transport Medium, mixed, and 500 µl transferred to the Truenat sample pretreatment buffer. Nucleic acids were extracted from the total volume using Trueprep AUTO v2 Sample Prep Device and Trueprep AUTO v2 Universal sample prep kit. For the alternative sample secondary outcome, a tongue swab was added directly to a tube containing a mid-turbinate nasal swab in 500 µl Transport Medium, and the full volume was similarly extracted on the Trueprep platform. Thereafter, 6 µl of extracted nucleic acid was dispensed into a microtube containing the freeze-dried PCR reagents. After allowing ~30–60 seconds for the dried PCR reagents to rehydrate with the nucleic acids from the sample, 6 μL of clear suspension was pipetted and dispensed into the reaction well of the Truenat MTB Ultima/COVID-19 chip. The chip was then inserted in the Truelab Real Time micro–PCR Analyzer for simultaneous TB and COVID-19 detection. Preclinical validation by the manufacturer evaluated successful combined sample processing for PCR inhibition and microbial inactivation for clinical safety. All testing was conducted within 24 hours of sample collection. Specimens were transported and stored at 2–8 °C until testing.

### Microbiological reference tests

The TB microbiological reference standard (MRS) for the primary outcome of TB detection from sputum (with Truenat MTB Ultima/COVID-19) was a composite of testing positive for any of sputum GeneXpert MTB/RIF Ultra (first day), and/or sputum MGIT or LJ culture (first and second day). In addition, as part of this study all sputum specimens were subjected to acid-fast bacillus smear microscopy to support results stratification.

The TB MRS for the secondary outcome of TB detection from sputum (with Truenat MTB Ultima/COVID-19) when compared to TB detection from sputum by the GeneXpert Ultra was sputum MGIT and LJ culture (first and second day).

The COVID-19 reference standard for the secondary outcome of COVID-19 detection from nasopharyngeal swab (with Truenat MTB Ultima/COVID-19) was a country approved RT-PCR (first day) - in South Africa this test was the Cepheid Xpert Xpress SARS-CoV-2 assay; in Uganda this was the Molbio COVID-19 assay; in Peru this was either the ThermoFisher TaqPath CoV-2 assay or Logix Smart ABC (Influenza A/B, SARS-COV-2) assay; in India this was the Q- line COVID-19 RT-qPCR assay. Each of these assays were approved by respective national regulatory authorities for use in diagnosis of COVID-19.

### Data and statistical analysis

The sample size target was determined based on an assumed sensitivity of 80% for the Truenat MTB Ultima/COVID-19 multiplex assay to detect MTB, and with a significance level of 5%, an expected precision of 7%, and 80% power to obtain the confidence interval. The study aimed to enroll an overall minimum of 270 participants with confirmed TB. The study protocol has been previously published [11].

Using an estimated TB prevalence of 20%, with ~10% of patients assumed to be lost to follow up, the total sample size required to obtain the primary objective was 1,480, with a minimum sample size target of 370 participants for each site. Through a protocol amendment, we expanded target enrolment up to 2000 participants, to allow satisfactory power to achieve the secondary objective of determining the diagnostic accuracy of the assay using an alternative sample of tongue and mid-turbinate nasal swab.

Descriptive statistics on patient characteristics and estimates of diagnostic accuracy stratified by site, sputum smear status, previous TB history and previous HIV status diagnosis was performed. Categorical variables are summarised as absolute numbers and relative (percent) frequencies. Continuous variables are summarised as medians with interquartile ranges (25th and 75th percentiles). Wilson’s score method was used to determine the sensitivity and specificity and presented as point estimates with two-sided 95% confidence intervals. The analysis was performed using SAS System (Version 9.4 or higher) or R statistical language (version 3.4.0 or higher) and Microsoft Excel 2017 (version 15.34 or higher).

## Results

**Fig 1:**
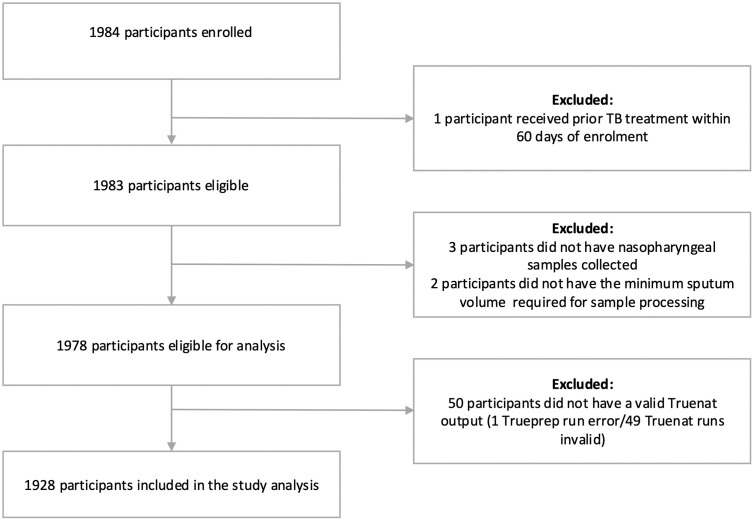
STARD diagram representation of participants enrolled, participants excluded, and participants included in the study analyses.

Between 31 August 2022 and 05 December 2023, 1,984 participants were enrolled, with 1,928 included in the analysis after exclusion of 56 participants (Fig 1). The median age of participants was 38 years (IQR 28–50 years), 47.6% (n/N = 918/1928) were female,18.6% (359/1928) had previous TB, and 14.9% (287/1928) reported or were diagnosed with HIV. The overall prevalence of diabetes and hypertension was 103/1926 (5.3%, 95% Confidence Interval (CI) 4.4-6.4%) and 114/1924 (5.9%, CI 5-7.1%) respectively. The overall prevalence of TB using the primary MRS for TB detection was 478/1928 (24.8%, CI 22.9-26.7%). Samples from all participants underwent sputum smear microscopy; of those who were microbiologically confirmed with TB by the primary MRS, 335/478 (70.1%) were sputum smear positive, ranging from 54/86 (62.8%) in Uganda to 125/153 (81.7%) in Peru. A total of 1588/1928 (82.4%) of all participants who underwent sputum smear microscopy were smear negative. During the study period, each country had a differing prevalence of COVID-19 based on country-specific waves. The overall prevalence of COVID-19 was 73/1910 (3.8%, CI 3.1-4.8%), with the prevalence of COVID-19 in those with confirmed TB at 22/470 (4.7%, CI 3.1-7%) (Table 1).

**Table 1:** Demographic, clinical and laboratory characteristics of participants in the study analysis.

	Total N = 1928 (%)	Uganda N = 611 (31.7%)	Peru N = 504 (26.1%)	South Africa N = 426 (22.1%)	India N = 387 (20.1%)
**Sex: n (%)**					
Male	1008 (52.3%)	295 (48.3%)	245 (48.6%)	238 (55.9%)	230 (59.4%)
Female	918 (47.6%)	316 (51.7%)	257 (51.0%)	188 (44.1%)	157 (40.6%)
Undifferentiated	2 (0.1%)	–	2 (0.4%)	–	–
**Age in years (IQR)**					
	38 (28.0-50.0)	32 (24.0-43.0)	41 (27.0-58.0)	38 (31.0-47.0)	46 (30.0-60.0)
**Previous history of TB n (%)**					
No	1559 (80.9%)	552 (90.3%)	383 (76.0%)	279 (65.5%)	345 (89.1%)
Yes	359 (18.6%)	55 (9.0%)	121 (24.0%)	147 (34.5%)	36 (9.3%)
Did not know	10 (0.5%)	4 (0.7%)	0 (0%)	0 (0%)	6 (1.6%)
**Previous diagnosis of HIV n (%)**					
No	1521 (78.9%)	455 (74.5%)	403 (80.0%)	278 (65.3%)	385 (99.5%)
Yes	287 (14.9%)	126 (20.6%)	14 (2.8%)	146 (34.3%)	1 (0.3%)
Did not know	120 (6.2%)	30 (4.9%)	87 (17.3%)	2 (0.5%)	1 (0.3%)
**Overall TB prevalence (N = 1920)**					
**n/N (%)** **(95% CI)**	476/1920 (24.8%) (22.9-26.8%)	86/606 (14.2%) (11.6-17.2%)	151/501 (30.1%) (26.3-34.3%)	130/426 (30.5%) (26.3-35.0%)	109/387 (28.2%) (23.9-32.8%)
**Microscopy Smear status for TB MRS positive participants (N = 478) n/N (%)**					
MRS-positive: Smear negative	143/478 (29.9%)	32/86 (37.2%)	28/153 (18.3)	48/130 (36.9%)	35/019 (32.1)
MRS-positive: Smear positive	335/478 (70.1%)	54/86 (62.8%)	125/153 (81.7%)	82/130 (63.1%)	74/109 (67.9%)
Scanty	58/335 (17.3%)	0/54 (0.0%)	40/125 (32.0%)	5/82 (6.1%)	13/74 (17.6%)
1+	111/335 (33.1%)	10/54 (18.5%)	31/125 (24.8%)	27/82 (32.9%)	43/74 (58.1%)
2+	78/335 (23.3%)	22/54 (40.7%)	25/125 (20.0%)	15/82 (18.3%)	16/74 (21.6%)
3+	88/335 (26.3%)	22/54 (40.7%)	29/125 (23.2%)	35/82 (42.7%)	2/74 (2.7%)
**Overall COVID-19 prevalence (N = 1910)**					
**n/N (%)** **(95% CI)**	73/1910 (3.8%) (3.1%-4.8%)	4/611 (0.7%) (0.3%-1.7%)	29/497 (5.8%) (4.1-8.3%)	21/415 (5.1%) (3.3-7.6%)	19/387 (4.9%) (3.2-7.5%)
**COVID-19 prevalence in TB MRS positive participants (N = 470)**					
**n/N (%)** **(95% CI)**	22/470 (4.7%) (3.1-7.0%)	1/86 (1.2%) (0.2-6.3%)	10/149 (6.7%) (3.7-11.9%)	6/126 (4.8%) (2.2-10.0%)	5/109 (4.6%) (2.0-10.3%)
**Overall Diabetes prevalence (N = 1926)**					
**n/N (%)** **(95% CI)**	103/1926 (5.3%) (4.4-6.4%)	15/611 (2.5%) (1.5-4.0%)	50/504 (9.9%) (7.6-12.8%)	28/424 (6.6%) (4.6-9.4%)	10/387 (2.6%) (1.4-4.7%)
**Overall Hypertensive prevalence (N = 1924)**					
**n/N (%)** **(95% CI)**	114/1924 (5.9%) (5.0-7.1%)	50/607 (8.2%) (6.3-10.7%)	19/504 (3.8%) (2.4-5.8%)	42/426 (9.9%) (7.4-13.1%)	3/387 (0.8%) (0.3%-2.3%)

N: Number of evaluated participants. n/N: Number of variable-positive participants/ number of evaluated participants. IQR: interquartile range; 95% CI: 95% Confidence Interval; TB MRS: TB primary microbiological reference standard of a composite of sputum GeneXpert MTB/RIF Ultra (first day), and sputum MGIT and LJ culture (first and second day)

During the diagnostic accuracy study, no Severe Adverse Events (SAEs) were reported.

### Diagnostic performance for TB diagnosis

The overall sensitivity of the Truenat MTB Ultima/COVID-19 assay for detection of TB from sputum was 380/476 (79.8%, CI 76.0-83.2%) when compared to the TB MRS. Sensitivity was better in sputum smear-positive participants at 310/333 (93.1%, CI 89.8-95.4%) (Table 2), and in India at 101/109, (92.7%, CI 86.2-96.2%) when compared to the TB MRS (Table 3). Overall specificity of the Truenat MTB Ultima/COVID-19 when compared to the TB MRS was 1428/1444 (98.9%, CI 98.2-99.3%) (Table 2).

**Table 2:** Diagnostic performance of Truenat MTB Ultima/COVID-19 for TB and COVID-19 detection among adults with TB symptoms compared to a composite reference of sputum GeneXpert Ultra, MGIT and LJ culture for TB, and a national site-specific RT-PCR for COVID-19, using (a) sputum and nasopharyngeal swab and (b) tongue and mid-turbinate swab.

	N	TP	FP	FN	TN	Sensitivity % (95% CI)	* Sensitivity % smear-positive (95% CI) (n/N)	*Sensitivity % smear-negative (95% CI) (n/N)	Specificity % (95% CI)
**(a) Sputum + nasopharyngeal swab**									
**MTB**	1920	380	16	96	1428	79.8% (76.0-83.2)	93.1% (89.8-95.4) (n = 310/333)	49.0% (40.9-57.1) (n = 70/143)	98.9% (98.2-99.3)
**COVID-19**	1902	47	15	26	1814	64.4% (52.9-74.4)	NA	NA	99.2% (98.7-99.5%)
**(b) Tongue swab + mid-turbinate nasal swab**									
**MTB**	837	94	7	111	625	45.9% (39.2-52.7)	63.7% (55.3-71.3% (n = 86/135)	10.9% (5.4-20.9) (n = 7/64)	98.9% (97.7-99.5)
**COVID-19**	832	5	2	7	818	41.7% (19.3-68.0)	NA	NA	99.8% (99.1-99.9)

N: Number of evaluated participant samples; TP: True Positive; FP: False Positive; FN: False Negative; TN: True Negative; 95% CI: 95% Confidence Interval; n/N: Number of test-positive participants/ Number of evaluated participants; NA: not applicable

*Smear-status of TB primary microbiological reference standard-positive participants. (TB primary microbiological reference standard is a composite of sputum Gene Xpert MTB/RIF Ultra (first day), and sputum MGIT and LJ culture (first and second day))

**Table 3:** Diagnostic performance of Truenat MTB Ultima/COVID-19 for TB detection among adults with TB symptoms compared to a composite reference of sputum GeneXpert Ultra, MGIT and LJ culture using (a) sputum and nasopharyngeal swab and (b) tongue and mid-turbinate nasal swabs stratified by previous HIV diagnosis, previous TB history and country.

	Number of evaluated participant samples	Sensitivity % (95% CI) (n/N)	Specificity % (95% CI) (n/N)
**(a) Sputum and nasopharyngeal swab**			
**Previous diagnosis of HIV**			
**Yes**	286	73.9% (62.5-82.8) (n = 51/69)	98.6% (96.0-99.5) (n = 214/217)
**No**	1515	81.3% (77.0-84.9) (n = 304/374)	99.0% (98.3-99.5) (n = 1130/1141)
**Previous history of TB**			
**Yes**	357	78.0% (69.3-84.7) (n = 85/109)	98.4% (95.9-99.4) (n = 244/248)
**No**	1553	80.3% (75.9-84.1) (n = 294/366)	99.0% (98.2-99.4) (n = 1175/1187)
**Country**			
**Uganda**	606	82.6% (73.2-89.1) (n = 71/86)	99.4% (98.3-99.8) (n = 517/520)
**Peru**	501	73.5% (66.0-79.9) (n = 111/151)	99.4% (97.9-99.8) (n = 348/350)
**South Africa**	426	74.6% (66.5-81.3) (n = 97/130)	98.6% (96.6-99.5) (n = 292/296)
**India**	387	92.7% (86.2-96.2) (n = 101/109)	97.5% (94.9-98.8) (n = 271/278)
**(b) Tongue swab and mid-turbinate nasal swab**			
**Previous diagnosis of HIV**			
**Yes**	161	42.4% (27.2-59.2) (n = 14/33)	100% (97.1-100) (n = 128/128)
**No**	612	49.0% (41.3-56.8) (n = 77/157)	98.7% (97.2-99.4) (n = 449/455)
**Previous history of TB**			
**Yes**	170	44.4% (30.9-58.8) (n = 20/45)	99.2% (95.6-99.9) (n = 124/125)
**No**	665	46.3% (38.7-54.0) (n = 74/160)	98.8% (97.4-99.5) (n = 499/505)
**Country**			
**Uganda**	356	51.6% (39.4-63.6) (n = 32/62)	99.0% (97.0-99.7) (n = 291/294)
**Peru**	225	33.8% (23.9-45.4) (n = 24/71)	98.7% (95.4-99.6) (n = 152/154)
**South Africa**	218	52.2% (40.5-63.7) (n = 35/67)	100% (97.5-100) (n = 151/151)
**India**	38	60.0% (23.1-88.2) (n = 3/5)	93.9% (80.4-98.3) (n = 31/33)

95% CI: 95% Confidence Interval; Sensitivity n/N: Number of index test-positive participants/ Number of composite reference test-positive participants; Specificity n/N: Number of index test-negative participants/ Number of composite reference test-negative participants

When compared to GeneXpert Ultra (sensitivity 387/419 (92.4%, CI 89.4-94.5%)), the Truenat MTB Ultima/COVID-19 ((sensitivity 347/419 (82.8%, CI 78.9-86.1%)) had a 9.5% (CI 6.5-13.1%) decreased sensitivity for TB detection when compared against sputum TB culture. The largest reduction in performance was in Peru, with a reduction in sensitivity by 17.9% (CI 12.0%-25.2%), with the other sites showing a reduction in sensitivity by 1.2 to 9.6%. The decreased sensitivity in sputum smear negative participants was also high, with a reduction of 19.6% (CI 9.4-30.1%) in sensitivity (Table 4).

**Table 4:** Diagnostic accuracy of Truenat MTB Ultima/COVID-19 multiplex compared to GeneXpert Ultra for TB detection among adults with TB symptoms against a reference standard of sputum TB culture.

	Sensitivity % (95% CI) (n/N)	Specificity% (95% CI) (n/N)
**Overall**	1906	
Truenat MTB Ultima/COVID-19	82.8% (78.9% to 86.1%) (n = 347/419)	96.7% (95.7% to 97.5%) (n = 1438/1487)
GeneXpert Ultra	92.4% (89.4% to 94.5%) (n = 387/419)	96.2% (95.1% to 97.1%) (n = 1431/1487)
Comparative: Truenat - Ultra	-9.5% (-13.1% to -6.5%)	0.5% (-0.4% to 1.3%)
**Country**		
Uganda	605	
Truenat MTB Ultima/COVID-19	87.8% (78.5% to 93.5%) (n = 65/74)	98.3% (96.8% to 99.1%) (n = 522/531)
GeneXpert Ultra	90.5% (81.7% to 95.3%) (n = 67/74)	97.7% (96.1% to 98.7%) (n = 519/531)
Comparative: Truenat - Ultra	-2.7% (-11.6% to 5.7%)	0.6% (-0.7% to 1.9%)
Peru	499	
Truenat MTB Ultima/COVID-19	75.2% (67.6% to 81.5%) (n = 109/145)	98.9% (97.1% to 99.6%) (n = 350/354)
GeneXpert Ultra	93.1% (87.8% to 96.2%) (n = 135/145)	98.3% (96.4% to 99.2%) (n = 348/354)
Comparative: Truenat - Ultra	-17.9% (-25.2% to -12.0%)	0.6% (-1.0% to 2.4%)
South Africa	417	
Truenat MTB Ultima/COVID-19	80.0% (71.8% to 86.3%) (n = 92/115)	97.0% (94.4% to 98.4%) (n = 293/302)
GeneXpert Ultra	89.6% (82.6% to 93.9%) (n = 103/115)	95.4% (92.4% to 97.2%) (n = 288/302)
Comparative: Truenat - Ultra	-9.6% (-16.3% to -5.4%)	1.7% (-0.8% to 4.4%)
India	385	
Truenat MTB Ultima/COVID-19	95.3% (88.5% to 98.2%) (n = 81/85)	91.0% (87.2% to 93.7%) (n = 273/300)
GeneXpert Ultra	96.5% (90.1% to 98.8%) (n = 82/85)	92.0% (88.4% to 94.6%) (n = 276/300)
Comparative: Truenat - Ultra	-1.2% (-7.9% to 5.2%)	-1.0% (-3.6% to 1.4%)
**Smear Status of TB sputum culture-positive participants**		
Smear Positive	322	
Truenat MTB Ultima/COVID-19	93.2% (89.9% to 95.4%) (n = 300/322)	NA
GeneXpert Ultra	99.7% (98.3% to 99.9%) (n = 321/322)	NA
Comparative: Truenat - Ultra	-6.5% (-9.9% to -4.0%)	NA
Smear Negative	97	
Truenat MTB Ultima/COVID-19	48.5% (38.8% to 58.3%) (n = 47/97)	NA
GeneXpert Ultra	68.0% (58.2% to 76.5%) (n = 66/97)	NA
Comparative: Truenat - Ultra	-19.6% (-30.1% to -9.4%)	NA
**Previous diagnosis of HIV**		
Previous positive HIV diagnosis	286	
Truenat MTB Ultima/COVID-19	78.7% (66.9% to 87.1%) (n = 48/61)	97.3% (94.3% to 98.8%) (n = 219/225)
GeneXpert Ultra	83.6% (72.4% to 90.8%) (n = 51/61)	96.4% (93.1% to 98.2%) (n = 217/225)
Comparative: Truenat - Ultra	-4.9% (-14.4% to 3.1%)	0.9% (-1.9% to 3.9%)
Previous negative HIV diagnosis	1502	
Truenat MTB Ultima/COVID-19	84.0% (79.7% to 87.6%) (n = 274/326)	96.5% (95.3% to 97.4%) (n = 1135/1176)
GeneXpert Ultra	94.5% (91.4% to 96.5%) (n = 308/326)	96.0% (94.7% to 97.0%) (n = 1129/1176)
Comparative: Truenat - Ultra	-10.4% (-14.6% to -6.8%)	0.5% (CI -0.4 to 1.5%)
**Previous TB history**		
History of previous TB (yes)	354	
Truenat MTB Ultima/COVID-19	85.4% (76.6% to 91.3%) (n = 76/89)	95.1% (91.8% to 97.1%) (n = 252/265)
GeneXpert Ultra	92.1% (84.6% to 96.1%) (n = 82/89)	92.5% (88.6% to 95.1%) (n = 245/265)
Comparative: Truenat - Ultra	-6.7% (-13.9% to -2.3%)	2.6% (-0.3% to 6.0%)
No history of prior TB	1542	
Truenat MTB Ultima/COVID-19	82.1% (77.6% to 85.8%) (n = 270/329)	97.0% (95.9% to 97.8%) (n = 1177/1213)
GeneXpert Ultra	92.4% (89.0% to 94.8%) (n = 304/329)	97.0% (95.9% to 97.8%) (n = 1177/1213)
Comparative: Truenat - Ultra	-10.3% (-14.5% to -6.6%)	0.0% (-0.8% to 0.8%)

95% CI: 95% Confidence Interval; Sensitivity n/N: Number of test-positive participant samples/ Number of reference test-positive participant samples; Specificity n/N: Number of test-negative participant samples/ Number of reference test-negative participant samples; NA: Not applicable

Comparison of the Truenat MTB Ultima/COVID-19 assay to the GeneXpert Ultra assay to detect MTB was done against a secondary reference standard of sputum TB culture alone. Sample sizes here reflect the number of participants where samples provided valid results for all tests of Truenat MTB Ultima/COVID-19, GeneXpert Ultra and sputum TB culture.

The overall sensitivity of the Truenat MTB Ultima/COVID-19 assay for detection of TB from tongue and mid-turbinate nasal swabs was 94/205 (45.9%, CI 39.2-52.7%) compared to the TB MRS. Overall specificity was 625/632 (98.9%, CI 97.7-99.5%). Sensitivity was higher but still poor in smear-positive participants (86/135, 63.7%, CI 55.3-71.3) (Table 2).

### Diagnostic performance for COVID-19 diagnosis

The overall sensitivity of the Truenat MTB Ultima/COVID-19 assay for detection of COVID-19 from nasopharyngeal swabs when compared to a national site-specific RT-PCR was 47/73 (64.4%, CI 52.9-74.4%) (Table 2). Performance was better in India with a sensitivity of 18/19 (94.7%, CI 75.4-99.1%) (S1 Table). Overall specificity was 1814/1829 (99.2%, CI 98.7-99.5%) (Table 2).

The overall sensitivity and specificity of the Truenat MTB Ultima/COVID-19 assay for detection of COVID-19 from tongue and mid-turbinate nasal swab was 5/12 (41.7%, CI 19.3-68.0%) and 818/820 (99.8%, CI 99.1-99.9%), respectively. (Table 2)

### Assay non-actionable results: error and invalid rates

S2 Table details the number of non-actionable results for each specimen type. Of 1,980 Trueprep assays run using the sputum plus nasopharyngeal swab method, 19 (0.96%) resulted in an error or no result, and 2 had missing data. When repeated, 18/19 (95%) resolved. Extracted DNA from 1,977 participants was run on the Truenat assay, with 1,705 (86.2%) providing valid results. The 271 samples with invalid or no results were repeated, with 215 (79.3%) resolving on repeat testing. In total, 1,920/1,977 (97%) samples tested yielded actionable results.

When testing the alternative sample of tongue swab plus mid-turbinate nasal swab, 3/852 (0.35%) Trueprep run samples were no result/error, and 3 (0.35%) were not done. Of the 3 samples repeated, all resolved. When tested on Truenat, 104/849 (12.25%) were invalid, and 7 (0.82%) were no result/error. Upon repeat Truenat testing, 99/111 (89.19%) resolved. In total, 837/849 (98.6%) of samples tested yielded actionable results.

## Discussion

The COVID-19 pandemic had a large negative impact on TB prevention and treatment [1,4–7]. Decades of progress made in TB control and elimination was set back, exposing the need for single diagnostic tests where TB testing is integrated with testing of other relevant and circulating respiratory illnesses capable of causing comparable clinical presentation and public detriment. New dual or multi-pathogen detection tests need to be accurate, rapid, affordable and accessible, particularly at primary healthcare centres with limited infrastructure and resources.

In this multi-centre diagnostic accuracy study in adults with signs and symptoms of TB, we determined that the multiplex Truenat MTB Ultima/COVID-19 assay was able to detect both MTB and SARS-CoV-2 in participants in a single assay. This study showed a sensitivity of 79.8% and specificity of 98.9% for detection of MTB when a sputum sample was mixed with a nasopharyngeal swab, and 64.4% sensitivity and 99.2% specificity for detection of COVID-19. Using an alternative sampling method of oral tongue swab mixed with a mid-turbinate nasal swab, sensitivity for both MTB and COVID-19 was lower, at 45.9% and 41.4% respectively, while maintaining high specificity. Although optimal diagnostic performance was not demonstrated, the potential and need for rapid development of tests that integrate TB diagnosis with the detection of other relevant respiratory infections is highlighted.

Traditional methods of TB diagnosis have limitations. The shift in practice from smear microscopy and culture toward molecular tests for TB diagnosis and detection of rifampicin resistance has significantly advanced the pathway toward TB control by providing rapid and accurate diagnosis of both [12–14]. To overcome limitations of the Xpert MTB/RIF and Ultra tests, the Truenat MTB, Truenat MTB Plus and Truenat MTB-RIF Dx chip-based PCR assays were developed to be run on the battery-operated, low-complexity, room-temperature-stable Truelab device as an alternative molecular assay to improve accessibility to TB diagnostics, particularly in resource-constrained settings where advanced diagnostic laboratories and skilled laboratory staff are scarce [12]. The Truenat MTB Ultima/COVID-19 chip was further developed to be run on the same Truelab device to facilitate both TB and COVID-19 detection using a single test.

This is not the first such study to show value of integrated TB and COVID-19 testing, but it is to our knowledge the first such study to show integrated testing for detection of both pathogens in a single multiplex assay. A prior study in Peru demonstrated the concept of concurrent TB and COVID-19 testing using a single sputum sample run across two separate assays on the same Xpert platform [15]. The authors showed the diagnostic yield of Xpert Xpress on sputum was moderate for COVID-19 (detected 67% of COVID-19 cases), and high for TB using Xpert Ultra (detected 96% of culture-confirmed TB cases). Despite this moderate performance using sputum for COVID-19, this study demonstrated the feasibility of combined and integrated testing.

In our study, the Truenat MTB Ultima/COVID-19 assay was developed to detect both MTB and COVID-19 with improved sensitivity in a single assay, using both sputum and swab samples together. Compared to Truenat MTB Plus, the Ultima chip newly includes both the *IS6110* and *IS1081* multi-copy gene targets to detect MTB, with a hypothetical improvement in sensitivity.

The overall performance of the Truenat MTB Ultima/COVID-19 assay using the combination of both sputum and a nasopharyngeal swab for the detection of MTB in this study was similar to prior reports for Truenat MTB Plus on sputum alone [16–18]. While one study showed increased sensitivity of the Truenat MTB Ultima assay for detection of TB, compared to MTB Plus, we failed to see the same enhanced performance here. The study by Abdulgader *et al.* also showed a matched decrease in specificity, so more evidence may be required to evaluate if the new Ultima chip has improved performance overall [19].

However, when compared specifically for the detection of MTB in sputum alone on Xpert Ultra, the Truenat MTB Ultima/COVID-19 assay had 9.5% lower sensitivity. This may be related to innate assay design differences, operational parameters or population variance. Indeed, comparative performance varied across sites (Table 4), with the biggest difference seen in Peru and South Africa and the lowest in India and Uganda, suggesting difference by site may be due to variation in the patient populations sampled, quality of samples collected, or laboratory flow and processing of samples. This variation occurred despite carefully controlling for standardised participant recruitment and inclusion, sampling techniques, specimen transport, processing and storage, assay conduct, and data entry. In addition extensive monitoring for quality through both regular remote and in-person site visits, detailed and standardised SOPs and training, recording and interrogating deviations, and data review and cleaning conducted before database lock and analysis occurred. Regardless of these stringent measures, we cannot rule out that subtle differences in processing and sample handling may have been responsible for performance variation seen between sites, highlighting the importance of reviewing new test performance across multiple settings.

Several studies have shown marginally lower sensitivity of Truenat MTB Plus compared to Xpert Ultra when using sputum alone, although all are challenged by small sample sizes and restricted geographies [18,19]. This heterogeneity in TB diagnostic performance has also been seen with the Xpert assays [13,14]. In our study, the lower sample input volume was a likely contributing factor to lowered sensitivity: the total volume of sputum that was added to the Truenat MTB Ultima/COVID-19 assay was reduced, as there was a dilution effect when mixing 500 ul of sputum with the transport medium and the sample preparation buffer, and the assay itself is restricted to only a 6ul purified DNA input volume. We attempted to overcome this limitation by using an alternative sample collection approach, which we expected to be more acceptable to participants and without a large trade off in overall performance. By evaluating a combined oral tongue swab with a mid-turbinate nasal swab, where there was no concern about an inherent dilution factor, we saw reduced sensitivity of both MTB and COVID-19. This was not surprising for MTB, as there are published studies suggesting lower bacterial load on tongue swabs compared to sputum [20–22]. But it was surprising for COVID-19 where reports have shown largely comparable performance on midturbinate nasal swabs and nasopharyngeal swabs [23–25]. Nevertheless, the low sample size for clinically confirmed COVID-19 cases in this study precludes clear outcomes for detection of COVID-19.

Overall, the performance of the Truenat MTB Ultima/COVID-19 for COVID-19 detection in this study was unexpectedly poor. In previous diagnostic accuracy studies from India, performance as a single test was reported as having 100% sensitivity and 99–100% specificity [26,27]. In our own laboratory analytical study conducted in India, the performance of the Truenat MTB Ultima/COVID-19 chip for the detection of COVID-19 on biobanked combined nasopharyngeal swab and sputum was excellent (sensitivity 100%) against a nationally approved PCR test. We acknowledge that findings may be of limited utility given that this component of the study was only performed in India with a limited sample size (n = 344) [28]. Nevertheless, it does suggest that the Truenat MTB Ultima/Covid-19 assay has excellent capacity to detect COVID-19, but that the sampling or population may have limitations.

The sensitivity of the Truenat MTB Ultima/COVID-19 for COVID-19 detection varied by site (48.3%-94.7%) and prevalence (0.7-5.9%) of COVID-19 (Table 4), with performance best in India, again highlighting, similar to TB diagnostic performance, the heterogeneity of results potentially being based on differences in populations, staff and site specific factors, as well as sample collection, laboratory flow and processing of samples. Other factors that may have also contributed to the assay’s poorer performance for COVID-19 detection in this study could be because the study was conducted later in the pandemic, with fewer people presenting for COVID-19 diagnosis (as demonstrated by the low overall COVID-19 prevalence), participants having lower viral load and shorter periods of viremia post COVID-19 vaccination [29], and because the study inclusion criteria were based on typical TB symptoms rather than typical COVID-19 symptoms.

As designed, this multiplex assay underperformed in this study. A limitation of this study is that we did not perform head-to-head evaluation of the multiplex assay against their respective individual single-plex assays for MTB with sputum or COVID-19 with a nasopharyngeal swab. While this assay is unlikely to have further clinical utility based on performance, it has demonstrated the potential of the rapid utilisation of newly developed multiplex diagnostic tools, particularly for respiratory infections that are a serious public health concern. Further learnings could be gained from optimising sample collection techniques and processing steps of mixed specimen samples.

## Conclusion

Although this study did not demonstrate optimal results for either TB (sensitivity was 79.8% and specificity was 98.9%) or COVID-19 (sensitivity 64.4%, specificity 99.2%) detection, it highlights the potential of rapid development and utilisation of tests that integrate TB diagnosis with diagnosis of other existing or new respiratory infections, mitigating the consequences of missed TB diagnosis particularly on a large scale like the COVID-19 pandemic did.

## Supporting information

S1 Checklist Inclusivity in Global Health Questionnaire. (DOCX)

S1 Table Diagnostic performance of Truenat MTB Ultima/COVID-19 for COVID-19 detection among adults with TB symptoms compared to a COVID-19 country-approved RT-PCR using (a) sputum and nasopharyngeal swab and (b) tongue and mid-turbinate swab stratified by country. (DOCX)

S2 Table Number and reasons for non-actionable results for a) sputum and nasopharyngeal swab samples and b) tongue and mid-turbinate swab samples using the Trueprep and Truenat MTB Ultima/COVID-19 assay. (DOCX)

S1 File COMBO Study Team listing. (XLSX)
