# Supplementary material for: Diagnostic accuracy study of the multiplex Truenat MTB Ultima/COVID-19 assay for simultaneous detection of Tuberculosis and SARS-CoV2 (COVID-19)
Source: PLOS Glob Public Health. 2026 Jun 5;6(6):e0005859. doi: 10.1371/journal.pgph.0005859 (PMC13241011; doi:10.1371/journal.pgph.0005859)
Supplement: S1 Table — (DOCX) [file pgph.0005859.s002.docx]

S1 Table: Diagnostic performance of Truenat MTB Ultima/COVID-19 for COVID-19 detection among adults with TB symptoms compared to a COVID-19 country-approved RT-PCR using (a) sputum and nasopharyngeal swab and (b) tongue and mid-turbinate swab stratified by country.

|  | **Number of cases in analysis** | **Sensitivity**  **% (95% CI)**  **(n/N)** | **Specificity**  **%(95% CI)**  **(n/N)** |
| --- | --- | --- | --- |
| 1. **Sputum and nasopharyngeal swab** | | | |
| **Uganda** | 606 | 75.0% (30.1-95.4)  (n=3/4) | 99.0% (97.8-99.5)  (n=596/602) |
| **Peru** | 494 | 48.3% (31.4-65.6)  (n=14/29) | 98.7% (97.2-99.4)  (n=459/465) |
| **South Africa** | 415 | 57.1% (36.5-75.5)  (n=12/21) | 99.5% (98.2-99.9)  (n=392/394) |
| **India** | 387 | 94.7% (75.4-99.1)  (n=18/19) | 99.7% (98.5-100)  (n=367/368) |
| 1. **Tongue and mid-turbinate nasal swab** | | | |
| **Uganda** | 356 | 100% (20.7-100)  (n=1/1) | 100% (98.9-100)  (n=355/355) |
| **Peru** | 224 | 33.3% (9.7-70.0)  (n=2/6) | 100% (98.3-100)  (n=218/218) |
| **South Africa** | 214 | 40.0% (11.8-76.9)  (n=2/5) | 99.0% (96.6-99.7)  (n=207/209) |
| **India** | 38 | NA  (n=0/0) | 100% (90.8-100)  (n=38/38) |

95% CI: 95% Confidence Interval; Sensitivity n/N: Number of index test-positive participants / Number of composite reference test-positive participants; Specificity n/N: Number of index test-negative participants / Number of composite reference test-negative participants; NA: not applicable
