# Supplementary material for: Diagnostic accuracy study of the multiplex Truenat MTB Ultima/COVID-19 assay for simultaneous detection of Tuberculosis and SARS-CoV2 (COVID-19)
Source: PLOS Glob Public Health. 2026 Jun 5;6(6):e0005859. doi: 10.1371/journal.pgph.0005859 (PMC13241011; doi:10.1371/journal.pgph.0005859)
Supplement: S2 Table — (DOCX) [file pgph.0005859.s003.docx]

S2 Table: Number and reasons for non-actionable results for a) sputum and nasopharyngeal swab samples and b) tongue and mid-turbinate swab samples using the Trueprep and Truenat MTB Ultima/COVID-19 assay

| **n/N (%)** | **Trueprep (initial)^1^** | **Trueprep (repeat)^2^** | **Truenat  (initial)^3^** | **Truenat (repeat)^4^** | **Overall actionable result** |
| --- | --- | --- | --- | --- | --- |
| **(a) Nasophayngeal swab + sputum** | | | | | |
| **Completed/Valid** | 1959/1980 (98.9%) | 18/19 (94.7%) | 1705/1977 (86.2%) | 215/271 (79.3%) | 1920/1977 (97.1%) |
| **Invalid** | - | - | 252/1977 (12.8%) | 56/271 (20.7%) |  |
| **No result/Error** | 19/1980 (0.96%) | 1/19 (5.26%) | 19/1977 (0.96%) | - |  |
| **Not done** | - | - | 1/1977 (0.05%) | - |  |
| **missing data** | 2/1980 (0.10%) | - | - | - |  |
| **(b) Mid-turbinate + tongue swab** | | | | | |
| **Completed/Valid** | 846/852 (99.3%) | 3/3 (100.0%) | 738/849 (86.9%) | 99/111 (89.2%) | 837/849 (98.6%) |
| **Invalid** | - | - | 104/849 (12.3%) | 12/111 (10.8%) |  |
| **No result/Error** | 3/852 (0.35%) | - | 7/849 (0.82%) | - |  |
| **Not done** | 3/852 (0.35%) | - | - | - |  |

n/N: Number of variable-positive participants/ number of evaluated participants.

^1^ 'Total' is the number of participants with both samples for a) sputum and nasopharyngeal swab and (b) tongue swab and mid-turbinate nasal swab received at the laboratory for testing

^2^ 'Total' is the number of participants with initial Trueprep yielding `No result/Error´

^3^  'Total' is the number of participants with Trueprep (either initial or repeat) being `Completed´

4 'Total' is the number of participants with initial Truenat yielding `Invalid´ or `No result/Error´
